# Resequencing Analyses Revealed Genetic Diversity and Selection Signatures during Rabbit Breeding and Improvement

**DOI:** 10.3390/genes15040433

**Published:** 2024-03-29

**Authors:** Kerui Xie, Chao Ning, Aiguo Yang, Qin Zhang, Dan Wang, Xinzhong Fan

**Affiliations:** 1 College of Animal Science and Veterinary Medicine, Shandong Agricultural University, Tai’an 271018, China; xiekerui666@163.com; 2 Shandong Provincial Key Laboratory of Animal Biotechnology and Disease Control and Prevention, Shandong Agricultural University, 61 Daizong Street, Tai’an 271018, China; ningchao@sdau.edu.cn (C.N.); qzhang@sdau.edu.cn (Q.Z.); 3 Key Laboratory of Efficient Utilization of Non-Grain Feed Resources (Co-Construction by Ministry and Province), Ministry of Agriculture and Rural Affairs, College of Animal Science and Veterinary Medicine, Shandong Agricultural University, Tai’an 271018, China

**Keywords:** rabbits, selection signatures, genetic analysis

## Abstract

Domestication has shaped the diverse characteristics of rabbits, including coat color, fur structure, body size, and various physiological traits. Utilizing whole-genome resequencing (DNBSEQ-T7), we analyzed the genetic diversity, population structure, and genomic selection across 180 rabbits from 17 distinct breeds to uncover the genetic basis of these traits. We conducted whole-genome sequencing on 17 rabbit breeds, identifying 17,430,184 high-quality SNPs and analyzing genomic diversity, patterns of genomic variation, population structure, and selection signatures related to coat color, coat structure, long hair, body size, reproductive capacity, and disease resistance. Through PCA and NJ tree analyses, distinct clusters emerged among Chinese indigenous rabbits, suggesting varied origins and domestication histories. Selective sweep testing pinpointed regions and genes linked to domestication and key morphological and economic traits, including those affecting coat color (*TYR*, *ASIP*), structure (*LIPH*), body size (*INSIG2*, *GLI3*), fertility (*EDNRA*, *SRD5A2*), heat stress adaptation (*PLCB1*), and immune response (*SEC31A*, *CD86*, *LAP3*). Our study identified key genomic signatures of selection related to traits such as coat color, fur structure, body size, and fertility; these findings highlight the genetic basis underlying phenotypic diversification in rabbits and have implications for breeding programs aiming to improve productive, reproductive, and adaptive traits. The detected genomic signatures of selection also provide insights into rabbit domestication and can aid conservation efforts for indigenous breeds.

## 1. Introduction

Rabbits have been domesticated for thousands of years [1]. The process can be divided into two stages: the first stage was from wild to domestic animals, and the second one is from domestic animals to landraces and even improved breeds. We identified genomic regions influenced by domestication in our previous study in which the genomes of wild rabbits (from the Iberian Peninsula and southern France) and domestic populations (from Europe and China) representing different geographic and genetic origins were compared. In this study, we wanted to explore the genetic diversity and genomic signature during the second stage, when the rabbits showed more distinct morphological and economic traits, and selective signatures were expected to be present in the genomes of different breeds due to adaptation to a diverse range of environments and specialized production systems during breeding and improvement.

Rabbit breeding holds significant global importance due to its contribution to food security, sustainable agriculture, and the development of new pharmaceuticals, with over 300 recognized rabbit breeds (https://www.fao.org/dad-is/dataexport/en/, accessed on 5 November 2023) worldwide reflecting a rich genetic diversity. This diversity provides an invaluable resource for genetic research, offering insights into genetic mechanisms underlying phenotypic variation, disease resistance, and adaptation to different environments. The extensive use of rabbits in genetic studies has also facilitated advancements in understanding complex genetic diseases in humans, making them a vital model organism in biomedical research [1,2]. There are at least 40 indigenous, cultivated, and recently introduced rabbit breeds in China, which are primarily distributed in Shandong, Sichuan, Henan, and Hebei [2]. Chinese indigenous rabbit breeds are characterized by their small body size and slow growth rate. However, they have excellent meat quality, strong resistance, and a high reproductive capacity. Some studies have been conducted on the genomic selection characteristics and major effect genes related to common traits in Chinese domestic rabbits. Coat color is the most prominent feature of domestic rabbits, and white rabbits are currently the most common and widely bred breed of domestic rabbit. *MC1R*, *ASIP*, *TYR*, and *KIT* are the important genes that influence coat color in rabbits [3,4,5]. The black spots on the coats of Checkered Giant rabbits are caused by mutations in *MC1R* [3]. *ASIP* controls the production of agoutis, which results in the gray-brown or gray coloration of dorsal fur [4]. Tyrosinase (TYR) is the rate-limiting enzyme required for the synthesis of melanin in melanosomes. Mutations in this gene can alter or impair the function of the encoded enzyme, leading to the occurrence of certain forms of albinism in domestic rabbits [6]. In addition, different breeds can also be distinguished by their hair structure and length. Natural mutations in the *LIPH* gene have been shown to be the cause of hair growth defects in humans and the Rex short-haired phenotype in rabbits [7]. Mutations in *FGF5*, a regulator of the hair cycle, can cause an inherited long-haired phenotype in various species, including domestic rabbits, resulting in an overall increase in hair length [8].

To date, high-density marker data, restriction-site associated DNA sequencing (RAD-seq), and whole-genome sequencing have been used to study selection signals in different rabbit breeds. For example, Carneiro et al. utilized whole-genome pooled sequencing to reveal the multi-gene basis of phenotypic changes during the domestication process [9]. Mohamad et al. identified genes associated with coat color, coat structure, and body size in meat and high-quality rabbit breeds based on high-density marker data [10]. Liu et al. explored the genomic resources of native Chinese indigenous rabbit breeds using RAD-seq technology and identified genes associated with melanin synthesis [11]. The development of next-generation sequencing (NGS) technology has utilized the high-throughput detection of Single Nucleotide Polymorphism (SNP) molecular markers, contributing to the study of animal genetics at the genomic level [12]. With the decrease in the cost of high-throughput sequencing, an increasing number of studies have started to utilize this technology for research on genomic diversity and selection features. In pigs, cattle, sheep, chickens, and other livestock and poultry, a large number of whole-genome sequencing studies have been used for the analysis of selection signals [13,14,15,16].

Our study bridges critical knowledge gaps by distinguishing between previously identified genes and uncovering new genetic markers associated with key adaptive traits in rabbits, including coat color, structure, long hair, body size, reproductive capacity, and disease resistance. By integrating findings from past research with our novel discoveries, we significantly enhance the understanding of rabbit genetics. This dual approach not only validates the contributions of earlier studies but also expands the genetic framework underlying rabbit adaptability and phenotypic diversity, offering a more comprehensive insight into their evolutionary development and potential for selective breeding enhancements. In the current study, the key research topic is as follows: to explore the genomic signatures underlying important morphological, production, and adaptive characteristics in Chinese rabbits during the breeding and improvement, we compared genomes of diverse domestic rabbit breeds to detect genetic diversity and positive selection traits that may explain their phenotypic variations.

## 2. Methods

### 2.1. Sample Collection and DNA Extraction

A total of 180 rabbits from 17 breeds were selected (Table 1); individuals were not related to each other (Table S1). Among them, 38 accessions were downloaded from the NCBI (PRJNA242290 [9] and PRJNA274594 [17]). Ear tissues from 142 rabbits were collected from China. Genomic DNA from each sample was isolated using a Qiagen MinElute Kit (Suzhou, China). Genomic DNA was utilized to create a paired-end library (PE150) with an insert size of approximately 350 bp, and was then sequenced on the DNBSEQ-T7 platform at a depth of 10X; MGI and Illumina sequencing platforms demonstrate comparable levels in sequencing quality, coverage, GC coverage, and variant accuracy. Compared to the Illumina platform, the MGI platform can be used in a wide range of genomics research areas at a lower cost [1,2].

### 2.2. Genotyping and Quality Control

1. Data Trimming and Quality Assessment: Whole-genome sequencing data were trimmed with fastp software (v0.23.4) [18] to remove adapters and low-quality reads. Additionally, FastQC (v0.11.9) (https://www.bioinformatics.babraham.ac.uk/projects/fastqc/, accessed on 15 September 2022) was used to assess the data quality. The clean data were mapped to the rabbit reference genome (GCF_000003625.3) [1] using BWA-mem (v0.7.17) [19]. The SAM files were converted to the BAM format and sorted using the SAMtools (v1.17) software.

2. Variant Detection and Filtering: To perform variant detection using the best GATK4 (v4.3.0.0) practices [20], the first step was to use the HaplotypeCaller module to process each individual and generate a gVCF file for each, which facilitated the subsequent analysis of additional samples. Next, the CombineGVCFs module was used to merge the gVCF files of all the samples, then the GenotypeGVCFs module was used to determine the SNP information for each individual. The VariantFiltration module was used to perform quality control (QC) on the SNPs with the following criteria: (a) QD < 2.0; (b) MQ < 40.0; (c) FS > 60.0; (d) SOR > 3.0; (e) MQRankSum < −12.5; and (f) ReadPosRankSum < −8.0. Finally, PLINK (v1.90) was used to filter the data based on the following criteria: genotype missing rate < 0.1, MAF > 0.05, and Hardy–Weinberg equilibrium (HWE) *p* value > 1 × 10^6^.

3. Annotation and Analysis: To annotate the filtered SNPs and identify variant functions, ANNOVAR (2019Oct24) software [21] was used. Additionally, the CMplot package [1] in the R environment was utilized to generate an SNP density plot, illustrating the distribution of SNPs across the whole genome.

### 2.3. Population Genomic Diversity Analyses

First, VCFtools (v0.1.16) [22] software was used to count the number of SNPs in each sample. The minor allele frequency (MAF), observed heterozygosity (Ho), and expected heterozygosity (He) of each breed were calculated with the PLINK (v1.90) software [23]. VCFtools (v0.1.16) software was used to calculate the nucleotide diversity (π) and population differentiation index (F_ST_) values. Runs of homozygosity (ROH) values for the autosomal SNPs in each individual were estimated using PLINK. Here, we defined ROH based on the following six criteria: a scanning window size of 50 SNPs; a minimum length of 100 kb for ROH regions; a required minimum density of 50 kb/SNP; a maximum gap of 100 kb between consecutive homozygous SNPs; maximums of one heterozygous genotype and five missing genotypes allowed within a scanned ROH region; and a threshold of 0.05 for all scanning windows containing SNPs. For assessment, ROH values were grouped into four categories based on length: 100–500 kb, 500–1000 kb, 1000–1500 kb, and >1500 kb. All parameter settings and evaluation classifications were based on previously published reports [24,25].

### 2.4. Population Genetic Analyses

In the principal component analysis (PCA), the input files (.bed, .bim, and .fam) were prepared using PLINK (v1.90) software. The genetic relationship matrix was calculated using GCTA (v 1.26.0) software [26] with the command “make-grm”. The calculated genetic relationship matrix was then used to compute the principal components using the command “-pca”. The neighbor-joining (NJ) phylogenetic tree was constructed using FastME 2.0 software [27] based on the genetic distance matrix calculated using VCF2Dis (v1.50) (https://github.com/BGI-shenzhen/VCF2Dis, accessed on 15 September 2022) and was visualized with Interactive Tree Of Life (iTOL) v4 [28]. In the population structure analysis, ADMIXTURE (v1.3.0) software [29] was used to infer the population structure. For each subpopulation (K = 2–8), the population clustering and individual ancestral composition were simulated.

### 2.5. Selection Signatures of Rabbit Genome

We classified rabbits into multiple groups based on their characteristics (Table 1). To explore the genomic resources related to coat color, we divided them into white and non-white rabbits. Within the non-white group, we further categorized them into black-furred rabbits and rabbits with different coat colors. To compare the body sizes of rabbits, we grouped the medium, large, and giant sizes together and compared them to a separate group consisting of small and dwarf rabbits. We compared Angora rabbits with all other types of rabbits to explore the genomic resources associated with hair growth and compared Rex rabbits with all other types of rabbits to explore the genomic resources associated with coat structure. In addition, we conducted grouping comparisons based on the resistance and reproductive capacity values of the rabbits.

We used the VCFtools (v0.1.16) software to perform a selection signature analysis on the genomes using the fixation index (F_ST_) and nucleotide diversity (π) methods. We utilized R scripts to generate relevant charts. Candidate genes were extracted from the scanning regions for further analysis.

### 2.6. Functional Enrichment

Annotated protein-coding genes were retrieved from the GFF file of OryCun2.0 NCBI using BEDtools (v.2.17) [30] to annotate the genomic regions that displayed selection signatures or suggestive selection signatures. Gene Ontology (GO) and Kyoto Encyclopedia of Genes and Genomes (KEGG) enrichment analyses were performed on the selected genes using DAVID [31]. These analyses were carried out to gain insights into the potential functions and biological pathways associated with the genes residing in the selected genomic regions exhibiting signals of positive selection. The annotation step helped identify the protein-coding genes within these regions of interest. Subsequently, the GO enrichment analysis elucidated the overrepresented biological processes, molecular functions, and cellular components related to the annotated gene set. The KEGG pathway enrichment analysis further revealed the significantly enriched metabolic or signaling pathways involving these genes. Together, these analyses facilitated the interpretation of the selective sweep results by providing functional contexts and clues about the potential phenotypic traits or adaptations that may have been targeted by positive selection during the breeding and domestication of different rabbit breeds.

## 3. Results

### 3.1. Sequencing and Identification of Single Nucleotide Polymorphisms

**Figure 1:**
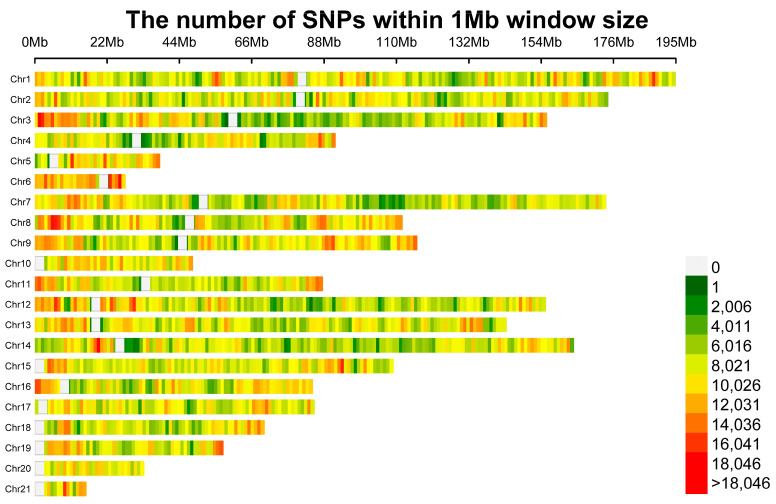
Distribution of SNPs on the chromosomes.

Eight rabbit breeds (REX, AN, KD, SCW, LWB, MXN, FJW, and JYS) were subjected to whole-genome sequencing, and their genotypes were jointly genotyped with publicly available genomes from nine foreign rabbit populations (EU, JW, NZW, and WHHL). In total, 187,512 million raw reads were generated, corresponding to 2.8 terabases. The BWA-MEM alignment tool was employed to map the sequencing reads to the *Oryctolagus cuniculus* reference genome (OryCun2.0), resulting in an average coverage of 15.3X. We detected 18,848,956 SNPs in the genome of 180 rabbits from 17 different breeds. After quality control, 17,430,184 high-quality SNPs were retained. The distribution of the SNPs on the chromosomes was plotted (Figure 1 and Table S1).

### 3.2. Genomic Diversity

The genomic diversity within each of the eleven breeds was assessed by estimating the nucleotide diversity (π), observed heterozygosity (Ho), expected heterozygosity (He), general minor allele frequency (MAF), and population differentiation index (F_ST_) (Table 2). The order of the recorded SNP counts for the different rabbit breeds was as follows: REX > LWB > JYS > AN > MXN > SCW > KD > NZW > FJW > WHHL > JW. REX, LWB, and JYS had the largest numbers of SNPs. Based on the SNP data, the nucleotide diversity of each breed was estimated. The nucleotide diversity ranged from 0.0014 to 0.0027, and REX, LWB, and JYS had the highest nucleotide diversity. The average MAF within the breeds ranged from 0.1001 to 0.237. All rabbits had the highest number of SNPs with an MAF lower than 0.05, and the lowest number of SNPs was located between 0.45 and 0.5. Among them, WHHL had the highest number of SNPs with a MAF lower than 0.05, indicating a higher prevalence of rare variants in this population. On the other hand, JYS exhibited the largest number of informative SNPs (MAF ≥ 0.45), suggesting a greater abundance of common variants in this population (Table S2). FJW had the lowest Ho value of 0.3043, while KD had the highest value of 0.3466. The He value ranged between 0.3109 in FJW and 0.3379 in NZW. The general F_ST_ values of the rabbits were between 0.1469 in JYS and 0.3102 in WHHL. Among them, the genetic differentiation was greatest between WHHL and JW (F_ST_ = 0.45), while it was the smallest between LWB and JYS (F_ST_ = 0.08) (Table S3).

### 3.3. Patterns of Genomic Variation

Runs of homozygosity (ROH) represent consecutive homozygous regions within the DNA sequences of diploid organisms [32]. To assess the ROH patterns in rabbits, we grouped ROH lengths into the following four size categories: 100–500 kb, 500–1000 kb, 1000–1500 kb, and >1500 kb (Table 3). The presence of longer ROH segments is indicative of close inbreeding, while shorter ROH segments reflect the influence of distant ancestors [33]. For most breeds, the majority of ROH lengths fell within the range of 100–500 kb. Notably, the JW and WHHL breeds exhibited the largest total ROH lengths. They are often used for experimental purposes and are subjected to a higher degree of artificial selection. In contrast, the JYS and LWB breeds displayed the shortest total ROH lengths, which are associated with their higher heterozygosity and genetic diversity.

### 3.4. Population Relationship and Structure

**Figure 2:**
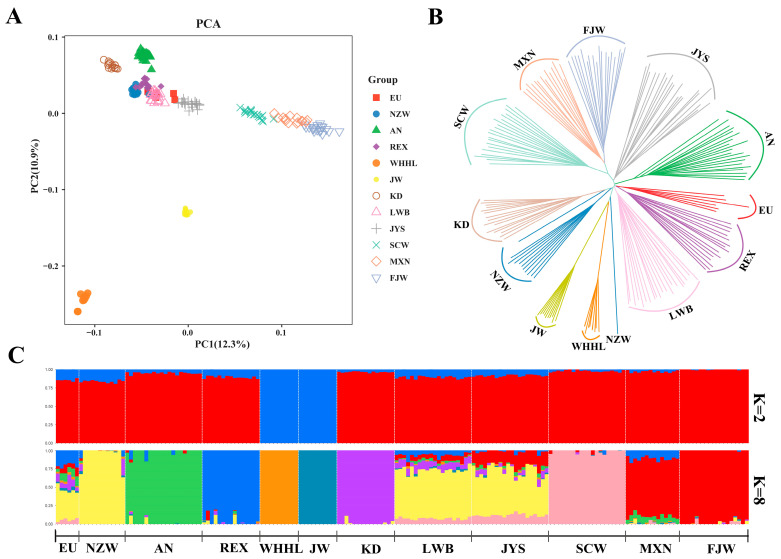
Results of population genomic analyses of 17 rabbit breeds: (**A**) PCA analysis; (**B**) neighbor-joining tree; (**C**) structure analysis.

To assess the genetic relationships and structures among the 11 rabbit breeds, PCA (Figure 2A), NJ tree (Figure 2B), and ADMIXTURE (Figure 2C and Figure S1) analyses were conducted. The first principal component explained 12.3% of the total variation and was used to visually depict the relationship between domestic rabbits from China (JYS, MXN, and FJW) and other regions. The second principal component explained 10.9% of the total variance and was used to visually distinguish JW and WHHL from the other breeds.

The NJ tree analysis revealed that Chinese indigenous rabbits (SCW, MXN, and FJW), meat rabbits (KD and NZW), Angora rabbits (AN), Japanese rabbits (JW and WHHL), and Rex rabbits (REX) are genetically distinct breeds. REX and AN exhibited less genetic distance to European domestic rabbits, aligning with their shared origins. LWB and JYS exhibited more genetic distance from other Chinese indigenous rabbits, and, notably, LWB’s genetic distance was closer to that of foreign breeds. The genetic distance between Japanese rabbits (JW and WHHL) and other domestic rabbits was greater, which was consistent with the results of the F_ST_ analysis and PCA.

To further analyze the population structure, we used ADMIXTURE to study the admixture of the populations, with K values ranging from 2 to 8. When K = 2, Japanese rabbits (JW and WHHL) had different genetic backgrounds from other rabbits, showing a relatively long genetic distance compared to other rabbits, which was consistent with the results of the PCA and the NJ tree. Based on cross-validation error, K = 8 was identified as the optimal number of gene clusters defining the population structure of the 17 rabbit breeds (Table S4). At K = 8, NZW, AN, REX, WHHL, JW, KD, SCW, and FJW had relatively homogeneous ancestral backgrounds, with only one major genetic ancestor. EU, LWB, and JYS had relatively complex ancestral backgrounds, with multiple genetic ancestors.

### 3.5. Genomic Signatures of Coat Color

**Figure 3:**
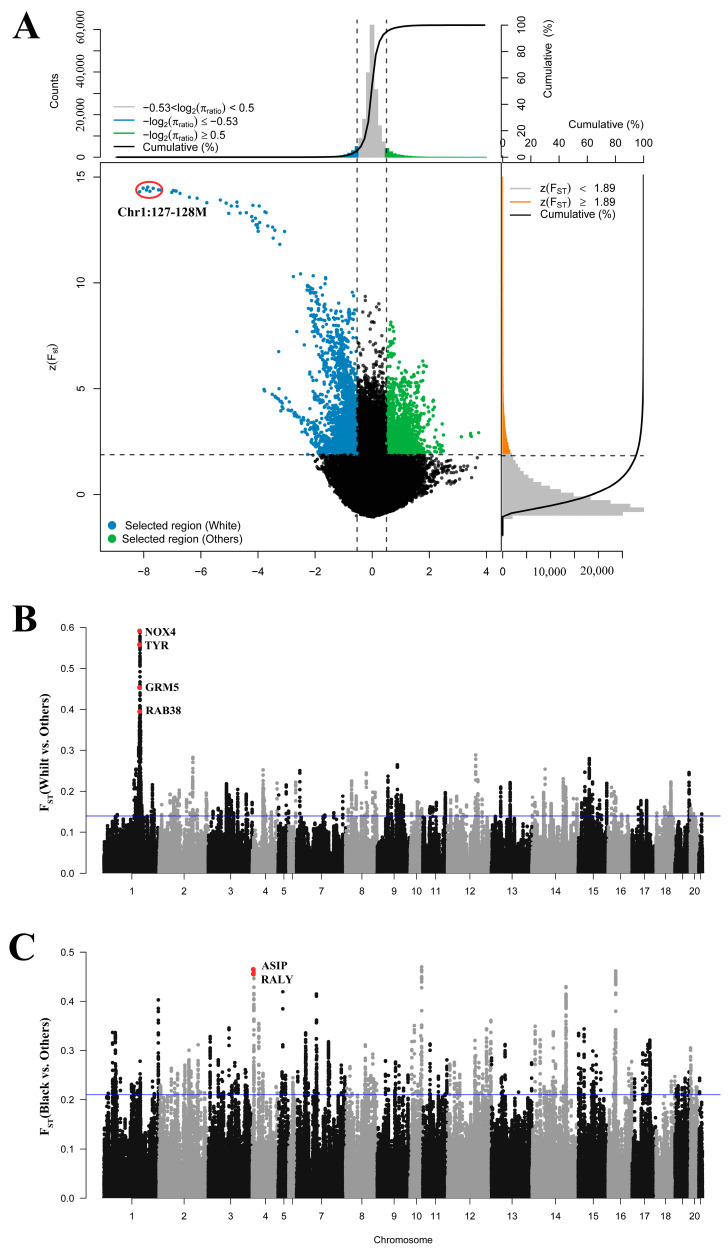
Selected regions between white-coat and non-white-coat rabbits. (**A**) Distribution of θπ ratios (white-coat/non-white-coat) and Z(F_ST_) values. (**B**) F_ST_ analysis based on genome-wide windows between white and non-white rabbits. (**C**) F_ST_ analysis based on genome-wide windows between black rabbits and other rabbits (excluding white-coat rabbits).

To detect genome-wide selection signatures, we calculated pairwise F_ST_ and θπ values in 50 kb sliding windows with a 10 kb step size between the white and non-white populations on autosomes. For each comparison, shared windows with F_ST_ and θπ values in the top 5% were considered as potential positive selection regions. Based on this method, a total of 2736 outlier windows were identified as selective sweep resgions in the whole-genome screening, annotating 1446 genes. We identified 58 GO-enriched terms and 9 KEGG pathways (Table S5). A strong selective region was discovered on chromosome 1 (Figure 3A). Four genes related to melanin synthesis and pigment deposition, including *TYR*, *GRM5*, *NOX4*, and *RAB38*, were found in this region (Figure 3B).

To further investigate the differences in coat color, we calculated the F_ST_ values between the black rabbits (MXN and LWB) and the other rabbits (JYS, French Lop, Dutch, Champagne, Belgian Hare, and Netherland Dwarf). A total of 1569 genes with selection signatures were obtained in the top 5% of the regions, and a total of 149 GO-enriched terms and 23 KEGG pathways were identified (Table S6). We found that *MC1R*, *ASIP,* and *RALY* are associated with melanin deposition and that *ASIP* and *RALY* have undergone strong selection (top 1%) (Figure 3C).

### 3.6. Signatures of Selection Detected for Coat Structure

**Figure 4:**
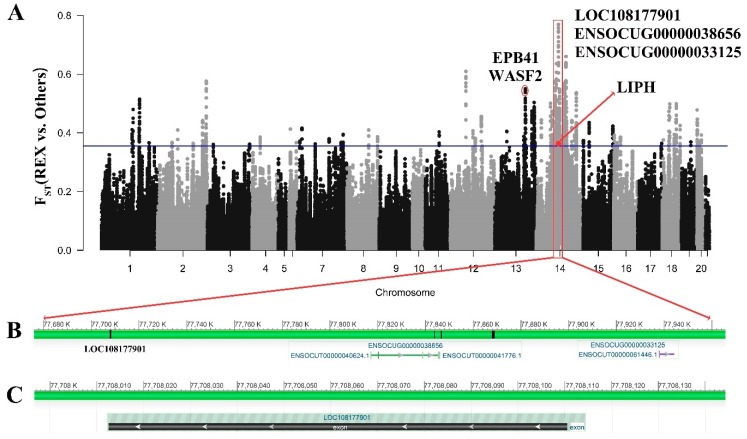
Selected regions between REX and other rabbits. (**A**) F_ST_ analysis based on genome-wide windows between REX and other rabbits (The red dots and red areas indicate the locations of genes). (**B**) The positions of the significant regions on the chromosomes. (**C**) LOC108177901 to a specific exon in the genome.

We calculated the F_ST_ values between REX and other populations and identified potential positive selection regions in the top 5% of shared windows with F_ST_ values greater than 0.22. A total of 1592 genes were annotated as selected, among which 385 genes exhibited strong selection signals (F_ST_ > 0.35, top 1%). In our study, we observed the *EPB41* and *WASF2* genes, which regulate the development of the epidermis. In addition, we identified a highly selected region on chromosome 14 in both Rex rabbits and other rabbits (Figure 4A). The selection intensity in this specific region was significantly higher compared to other regions on the same chromosome. Within this region, we discovered one gene (LOC1081770901) and two lncRNAs (Figure 4B,C) that could potentially play a role in determining the coat structure.

### 3.7. Signatures of Selection Detected for Long Hair

**Figure 5:**
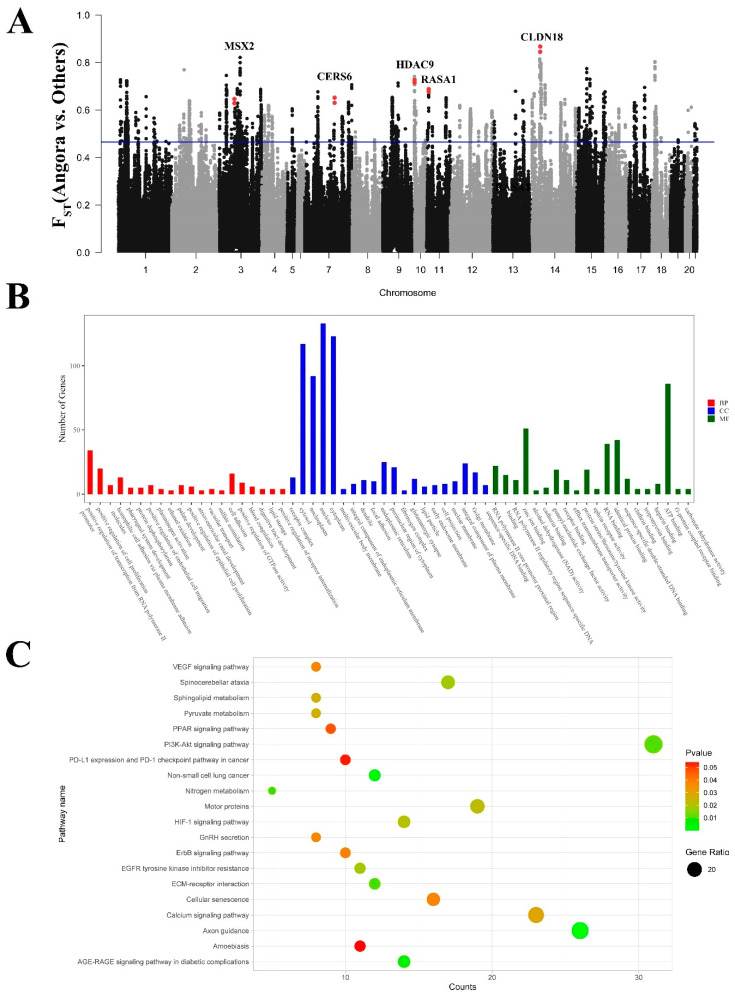
GO classification and KEGG enrichment of the selected genes between Angora rabbits and other rabbits. (**A**) F_ST_ analysis based on genome-wide windows between Angora rabbits and other rabbits (The red dots indicate the positions of genes and F_ST_ values). (**B**) GO classification of the selected genes. (**C**) KEGG enrichment of the selected genes.

Angora rabbits are notable for their long hair, which distinguishes them from other domestic rabbit breeds. Additionally, their hair growth rate is significantly higher than that of other domestic rabbits. To investigate selection signals associated with hair length and growth in Angora rabbits compared to other domestic rabbit breeds, we conducted a sliding window analysis to calculate F_ST_ values (Figure 5A). We identified regions displaying high F_ST_ values (top 5%) and annotated them to 1414 genes. Subsequently, a DAVID enrichment analysis was performed to determine the GO terms and KEGG pathways associated with these genes, revealing a total of 120 GO terms and 33 KEGG pathways (Figure 5B,C). In our enrichment analysis, we identified important pathways related to the regulation of hair follicle development and growth, such as Jak-STAT and PI3K-Akt (Table S7). In the top 1% F_ST_ region, we identified five genes (*MSX2*, *CERS6*, *HDAC9*, *RASA1*, and *CLDN18*) that exhibited a strong association with hair development (Figure 5A).

### 3.8. Signatures of Selection Detected for Body Size

**Figure 6:**
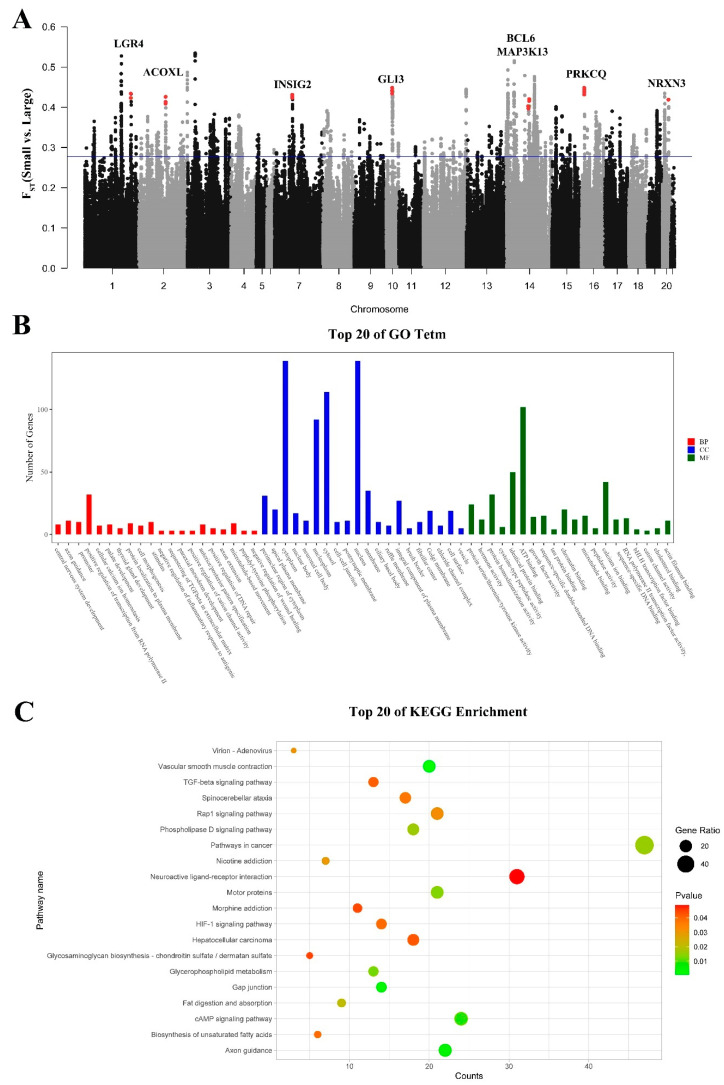
GO classification and KEGG enrichment of the selected genes between small-bodied and large-bodied rabbits. (**A**) F_ST_ analysis based on genome-wide windows between small-bodied and large-bodied rabbits (The red dots indicate the positions of genes and FST values). (**B**) GO classification of the selected genes. (**C**) KEGG enrichment of the selected genes.

To detect selection signatures associated with the growth rate and body size in rabbits, we scanned the rabbit genome for regions with high coefficients of nucleotide differentiation (F_ST_) between the populations of large-bodied (Belgian Hare, Champagne, Flemish Giant, French Lop, NZW, AN, REX, WHHL, JW, KD, and LWB) and small-bodied (Netherland Dwarf, Dutch, JYS, SCW, MXN, and FJW) rabbits based on 50 kb sliding windows (Figure 6A). We identified 1555 genes with F_ST_ scores in the top 5%. A GO classification and a KEGG enrichment analysis were conducted on the genes exhibiting selection signatures in rabbits (Figure 6B,C). We found many terms related to digestion, metabolism, and growth (Table S8). We identified eight genes closely associated with growth, development, and body size in the top 1% F_ST_ region (Table 4). *INSIG2*, *PRKCQ*, *GLI3*, and *LGR4* were associated with weight and body size. *NRXN3* and *ACOXL* were related to obesity. *MAP3K13* and *BCL6* were associated with growth and development. Additionally, we found that *COL21A1*, *MSRA* [10], and *IRS1* [34] were associated with growth traits in rabbits.

### 3.9. Signatures of Selection Detected for Reproductive Capacity

**Figure 7:**
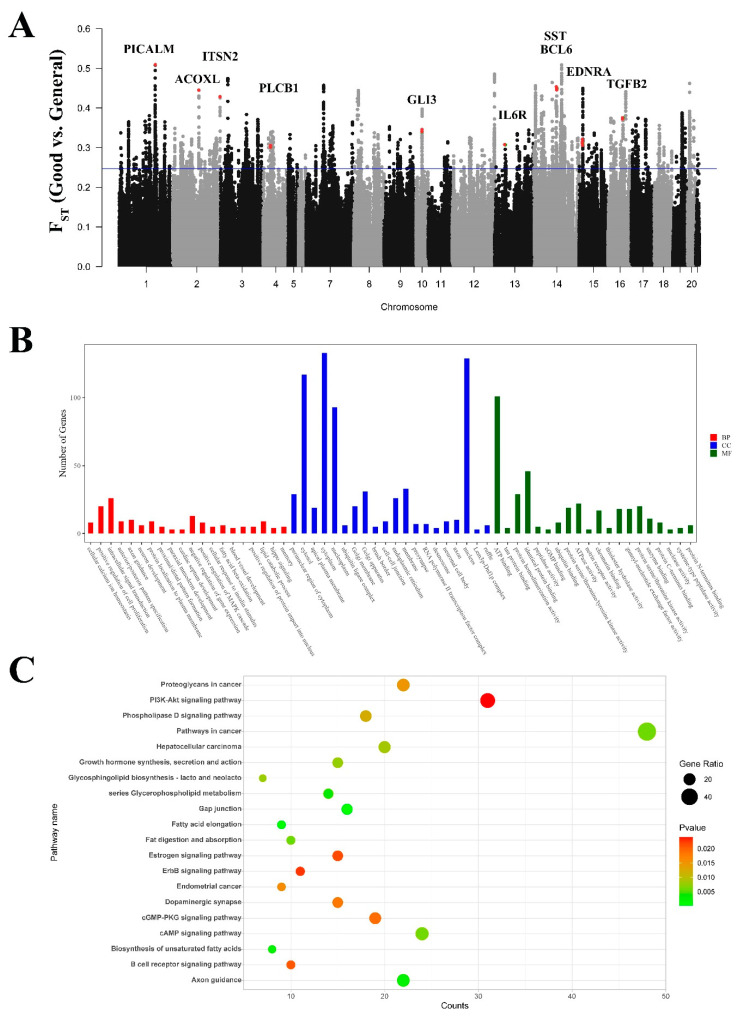
GO classification and KEGG enrichment of the selected genes between rabbits with good reproductive capacity and rabbits with general reproductive capacity. (**A**) F_ST_ analysis based on genome-wide windows between good reproductive and general reproductive rabbits (The red dots indicate the positions of genes and FST values). (**B**) GO classification of the selected genes. (**C**) KEGG enrichment of the selected genes.

The focus of the domestic rabbit breeding program is on traits related to reproductive efficiency, such as reproductive capacity. The influence of different breeds results in variation in the onset of sexual maturity and in the pregnancy rate in domestic rabbits. We scanned the rabbit genome for regions with high coefficients of nucleotide differentiation between the populations of rabbits with good reproductive capacity (JYS, SCW, MXN, and FJW) and general reproductive capacity (NZW, AN, REX, WHHL, JW, KD, and LWB) based on 50 kb sliding windows (Figure 7A). We identified 1495 genes with F_ST_ scores in the top 5%, and a GO classification and a KEGG enrichment analysis were conducted on the genes exhibiting selection signatures in the rabbits (Figure 7B,C), revealing that many of them were associated with hormone-related and gonad development pathways (Table S9). Among them, we identified nine highly selected genes (top 1%) that were closely associated with precocious sexual maturity (*PLCB1*), gonadal development (*PLCB1*, *GLI3*, and *TGFB2*), or fertility (*SST* and *EDNRA*) (Table 5).

### 3.10. Signatures of Selection Detected for Resistance

**Figure 8:**
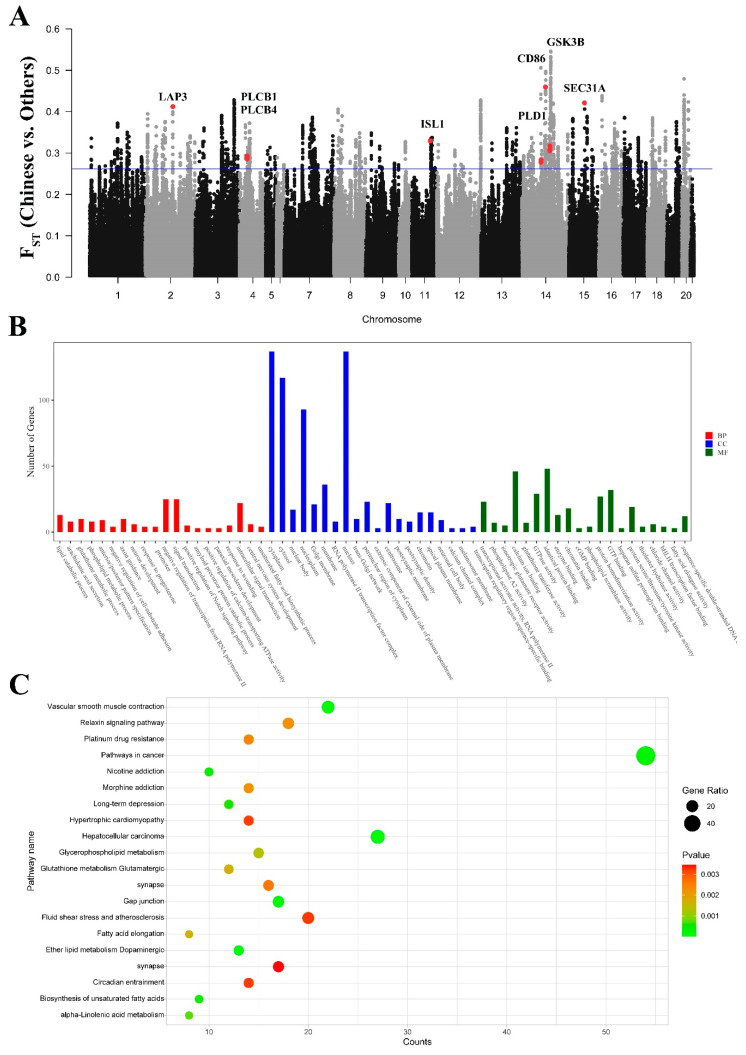
GO classification and KEGG enrichment of the selected genes between Chinese indigenous rabbits and other rabbits. (**A**) F_ST_ analysis based on genome-wide windows between Chinese indigenous rabbits and other rabbits (The red dots indicate the positions of genes and F_ST_ values). (**B**) GO classification of the selected genes. (**C**) KEGG enrichment of the selected genes.

A comparison of Chinese indigenous rabbits (JYS, SCW, FJW, and MXN) with other breeds revealed numerous disease-related genes. In the GO and KEGG enrichment analyses, we identified a significant number of entries related to diseases and macrophages (Figure 8B,C and Table S10). This highlighted their superior environmental adaptability and disease resistance. Within the strong selection region (Figure 8A), we identified seven genes closely associated with resistance (Table 6), including genes related to environmental adaptability (*PLCB1*, *GSK3B*, and *ISL1*) and immune ability (*PLD1*, *LAP3*, *CD86*, and *SEC31A*).

## 4. Discussion

In this study, we utilized whole-genome resequencing to obtain a vast number of genomic variations, elucidating the genetic backgrounds of different rabbits. The results showed that JYS and LWB exhibited the highest genetic diversity. This may be the result of hybridization with other breeds [11,72]. The genetic diversity of JW and WHHL rabbits was significantly lower than that of other rabbits, which is consistent with a recent report [17]. JW and WHHL were genetically distant from other rabbit breeds, which is consistent with their breeding history and may be related to the process of artificial selection [17].

Through selective sweep analyses, we identified several selective signatures in the rabbit genome. Some of the results we obtained highlighted genes previously identified to influence coat color (*TYR*) [11], pigmentation-related traits (*ASIP*) [4,10], and coat structure (*LIPH*) [7,10,73]. This demonstrates that the methods we employed are capable of capturing recent selective features. We found strongly selected regions on the genome in both the coat color and coat structure variations. These regions contain key genes that affect the color (*TYR*) and structure (*LIPH*) of rabbits’ coats.

*TYR* is a crucial gene in the process of melanin formation, and its variations lead to allelic series of white-coat-color loci [5], highlighting the breed-specific characteristics in this region. Variants located upstream of the *GRM5* gene have independent effects on pigmentation and may potentially regulate the expression of *TYR* [74]. The expression of *NOX4* can inhibit the expression of the tyrosinase gene, thereby reducing the production of melanin [75]. *RAB38* regulates the transport of melanogenic enzymes, particularly tyrosinase, from the trans-Golgi network (TGN) to melanosomes [76]. *NOX4*, *GRM5*, and *RAB38* have not been reported to have an impact on coat color in rabbits to date, but they all have the potential to influence the expression of tyrosinase genes [74,75,76], which control fur color in domestic rabbits.

*ASIP* is a widely studied pigmentation gene that plays an important role in melanin synthesis and is associated with the darkness or lightness of an animal’s coat color [77,78,79]. *RALY* and *ASIP* contribute to the number of facial pigmented spots acquired during the aging process through pathways independent of basal melanin production [80]. ASIP and RALY can also influence the coat color of horses and the deposition of facial pigmentation in humans [79,80]. They are important candidate genes that determine variations in coat color in domestic rabbits.

In the upstream region of *LIPH*, we observed intense selection acting on the unnamed genes and the region where long non-coding RNAs are located. The variation in coat color and structure of rabbits may be due to a mutation in a linked region. Our research has revealed the close association of *LIPH*, *FPB41*, and *WASF2* with hair follicle development. *LIPH* specifically influences the cortical structure impacted by the REX locus [7,73]. *EPB41* is closely linked to defects in hair follicle structure and function, as well as skin lesions and tumor development [81]. Additionally, *WASF2* acts as a critical regulator of epidermal shape and growth [82].

Furthermore, we also identified genes related to hair growth and hair follicle development (*MSX2*, *CERS6*, *HDAC9*, *RASA1*, and *CLDN18*) in the selection signals of Angora rabbits, which may provide new insights into the development of hair. HDAC gene family members play a significant role in modulating the structure of chromatin and act as crucial regulatory factors in gene transcription. They are closely associated with human baldness [83]. *MSX2* is involved in the processes of hair shaft differentiation and hair follicle neogenesis [84,85]. *CLDN18* plays a critical role in the differentiation process of the epidermis and hair follicles [86]. *RASA1* promotes the proliferation of goat hair follicle stem cells and inhibits cell apoptosis [8].

The complexity of the selection signatures observed in comparisons between small-, medium-, and large-sized rabbit breeds indicates that a multitude of genes influencing growth and development are involved in determining rabbit body size and productive performance. This finding is similar to reports in other mammals. *INSIG2*, *GLI3*, and *NRXN3* are known to affect human body mass index and height [35,38,47]. *LGR4* and *PRKCQ* have been found to be closely associated with body weight in studies of pigs and humans [44,49,50]. These are potential candidate genes that may contribute to the differentiation of body size in domestic rabbits.

In the exploration of genetic selection signals among rabbits with diverse reproductive capabilities, we have pinpointed several genes that play pivotal roles in reproductive traits, the development of sexual glands, and the onset of sexual maturity. These genes include SST, which is known to modulate the expression of genes crucial for fertility, as well as the maturation of oocytes and the development of embryos, highlighting its significant role in reproductive success [56]. EDNRA and ACOXL have been identified as potential markers for fertility in cattle [2], suggesting their broader importance in reproductive biology across species. These genes are implicated in essential reproductive processes, with EDNRA involved in the endothelin signaling pathway that influences vascular functions and follicular development, and ACOXL potentially linked to metabolic pathways critical for reproductive energy demands. GLI3 and TGFB2 are integral to the development of sexual glands [53,54,58], with GLI3 affecting the Sonic Hedgehog signaling pathway crucial for organogenesis, including gonadal development, and TGFB2 part of the transformative growth factor-beta pathway, known for its role in cellular differentiation and reproductive organ development. Lastly, PLCB1 has been associated with central precocious puberty and is crucial for the development of external genitalia, underlining its significance in reproductive maturity [51,52]. These discoveries not only shed light on the genetic underpinnings of reproductive traits in rabbits but also resonate with known mechanisms in other species, suggesting a conserved genetic framework underlying reproductive biology. This enhanced understanding opens avenues for further research into reproductive health and fertility across species, offering potential strategies for managing fertility and understanding reproductive disorders.

The identification of genes linked to environmental adaptation and immune responses in Chinese indigenous rabbits, as compared to foreign domestic rabbits, holds significant practical implications for adaptability and health across diverse environmental conditions. Zhang et al., through their study on the genetic variation in resistance to bacterial infection in growing meat rabbits, found that the incidence and mortality rates of bacterial infections in Chinese indigenous rabbits were lower than those in California rabbits, Belgian rabbits, and Chinchilla rabbits [1]. Chen et al., through their research on the nonspecific disease resistance characteristics of Chinese local domestic rabbits, discovered that they possess good nonspecific disease resistance [1,2]. Specifically, genes such as *PLCB1*, *LAP3*, *SEC31A*, *CD86*, *IL6R*, *ISL1*, *GSK3B*, and *PLD1* play pivotal roles in biological processes crucial for survival and adaptation. For instance, PLCB1 is integral to intracellular signal transduction, facilitating responses to a variety of extracellular signals. Its association with heat tolerance in other species suggests its potential in aiding Chinese indigenous rabbits to adapt to warmer climates [62,63]. Similarly, ISL1, which is crucial for heart development, might contribute to the high-altitude adaptability seen in some rabbit populations, mirroring findings in cashmere goats [65,66]. GSK3B’s involvement in hypoxic condition adaptation suggests that rabbits with this gene could better withstand low-oxygen environments [64]. *PLD1* plays a pivotal role in the autophagy process [67,87] and acts synergistically as a virulence factor, thereby significantly contributing to the invasion of host cells [68]. *LAP3*, *CD86,* and *SEC31A* play important roles in the immune system [69,70,71]. These genetic traits not only highlight the potential for selective breeding programs to enhance environmental resilience and disease resistance in rabbits but also underscore the importance of conserving genetic diversity within indigenous populations. Furthermore, understanding these genetic mechanisms offers a valuable model for biomedical research, potentially advancing our knowledge of human adaptability and immune response, and paving the way for novel therapeutic strategies.

Overall, the study of these genes not only sheds light on the genetic foundations of adaptability and health in Chinese indigenous rabbits but also opens avenues for practical applications in breeding, conservation, and biomedical research, emphasizing the critical role of genetic diversity in the resilience of animal populations to environmental and health challenges.

This study, while offering valuable insights into the genetic diversity and specific genes associated with adaptability, health, and phenotypic traits in rabbits, has its limitations, including the scope of genetic diversity covered, direct correlation between genetic markers and phenotypes, and the consideration of environmental interactions. Future research could address these limitations by expanding the genetic sampling across more rabbit breeds and wild populations, conducting functional genomic studies to elucidate the mechanisms behind gene-trait associations, exploring gene-environment interactions, and undertaking longitudinal studies to observe genetic changes over time. Such research endeavors would not only deepen our understanding of rabbit genetics but also enhance breeding programs and conservation efforts, and provide broader insights into evolutionary biology and genetics.

## 5. Conclusions

In our comprehensive exploration of the genetic diversity and population structure among Chinese indigenous rabbit breeds and foreign breeds through whole-genome resequencing, we discovered that the LWB, JYS, and REX breeds exhibit notably higher genetic diversity compared to others. Specifically, LWB and JYS breeds are distinguished by their multiple genetic ancestries, indicating a rich and complex genetic background that contributes to their unique characteristics. Further analysis revealed that a significant portion of the genetic ancestry of LWB and JYS breeds comes from the New Zealand White (NZW) rabbits, with a smaller contribution from the Fujian White (FJW) and other domestic rabbit breeds. This mixture of ancestries suggests that LWB and JYS rabbits have inherited a broad range of genetic traits, which may account for their distinct phenotypes and adaptabilities. The presence of NZW ancestry in particular could be linked to traits such as size, growth rate, and fur quality, while the influence from FJW and other breeds may add to their genetic diversity, potentially enhancing their resilience and adaptability to various environments. Our investigation into the selection signatures across diverse rabbit populations has unveiled multiple regions within the genome that show significant selection signals, particularly in traits related to coat color, coat structure, and hair growth. These findings not only enrich the existing pool of candidate genes associated with these traits but also highlight specific genes under strong selection in Chinese indigenous rabbits, underscoring their potential role in defining the breed’s distinctive features.

Building on these insights, we recommend future breeding endeavors to focus on leveraging the identified genetic diversity and selection signatures to enhance breed characteristics such as coat quality, color, and body size. Breeders should consider the unique genetic attributes and ancestral components of the LWB, JYS, and REX breeds when designing breeding programs, aiming to preserve or enhance these valuable traits. Additionally, the strong selection signals identified in Chinese indigenous rabbits offer a promising avenue for developing breeds with enhanced adaptability and distinct phenotypic traits.

Conclusively, our findings lay a foundational stone for the future of rabbit breeding, conservation efforts, and genetic research. By illuminating the genetic underpinnings that contribute to the diversity and distinctiveness of Chinese indigenous and foreign rabbit breeds, this study not only aids in the conservation of these breeds but also paves the way for informed breeding strategies. The insights gained from our research hold the potential to significantly influence the direction of future breeding practices, ensuring the preservation of genetic diversity and the enhancement of desirable traits in rabbit populations worldwide.

## Figures and Tables

**Table 1:** Details of the analyzed breeds and animals.

Breed	Acronym	Number of Animals ^1^	Coat Color ^2^	Body Size	Reproductive Capacity ^3^	Resistance ^4^
Netherland Dwarf	EU	14 (pool)	Black and White	Dwarf	-	-
Belgian Hare	EU	17 (pool)	Brownish	Medium	-	-
Champagne d’Argent	EU	16 (pool)	Argent	Large	-	-
Dutch	EU	13 (pool)	Black and White	Dwarf	-	-
Flemish Giant	EU	18 (pool)	Linen	Giant	-	-
French Lop	EU	20 (pool)	Brownish	Large	-	-
New Zealand White rabbit	NZW	12 (individual)	White	Medium	General	General
Angora rabbit	AN	20 (individual)	White	Large	General	General
Rex rabbit	REX	15 (individual)	White	Large	General	General
Watanabe heritable hyperlipidemic	WHHL	10 (individual)	White	Medium	General	General
Japanese Large-ear White rabbit	JW	10 (individual)	White	Medium	General	General
Kangda meat rabbit	KD	15 (individual)	White	Large	General	General
Laiwu Black rabbit	LWB	20 (individual)	Black	Medium	General	-
Jiuyishan rabbit	JYS	20 (individual)	Gray	Small	Good	Good
Sichuan White rabbit	SCW	20 (individual)	White	Small	Good	Good
Minxinan Black rabbit	MXN	14 (individual)	Black	Small	Good	Good
Fujian White rabbit	FJW	18 (individual)	White	Small	Good	Good

^1^ Pool = whole-genome resequencing of pooled samples; individual = whole-genome resequencing of individual sample. ^2^ Dutch and Netherland Dwarf rabbits have black ears and rumps, bands of white from the tops of their shoulders to their bellies, white legs, and wedges of white fur running up the fronts of their faces, which are called the ‘white blaze’. ^3^ Chinese indigenous rabbits generally reach sexual maturity about 30–40 days earlier than foreign breeds, and they have a conception rate that is 10–20% higher [2]. ^4^ The infection rates of respiratory diseases and fungal skin diseases in Chinese indigenous rabbits are significantly lower than those in introduced rabbits or hybrid-bred meat rabbits or fur rabbits [2].

**Table 2:** Overview of the investigated rabbit breeds and related population parameters.

Breed	N	MAF	π	Ho	He	F_ST_
AN	6,823,612	0.2054	0.0024	0.3189	0.3182	0.2023
FJW	6,390,537	0.1916	0.0023	0.3043	0.3109	0.1961
JW	5,081,336	0.1105	0.0015	0.3073	0.3356	0.2909
JYS	7,079,312	0.2307	0.0027	0.3345	0.3317	0.1469
KD	6,601,780	0.1911	0.0024	0.3296	0.3157	0.2090
LWB	7,080,476	0.2298	0.0027	0.3351	0.3334	0.1505
MXN	6,801,543	0.2068	0.0025	0.3332	0.3250	0.1844
NZW	6,450,438	0.2031	0.0025	0.3340	0.3379	0.1855
REX	7,094,777	0.2242	0.0027	0.3321	0.3349	0.1707
SCW	6,706,715	0.2076	0.0025	0.3214	0.3263	0.1848
WHHL	5,132,820	0.1001	0.0014	0.3122	0.3179	0.3102

N: number of SNPs; MAF: general minor allele frequency; π: nucleotide diversity; Ho: observed heterozygosity; He: expected heterozygosity; F_ST_: population differentiation index.

**Table 3:** Statistical analysis of the numbers of ROH fragments of different lengths between the rabbit populations.

Breed	100–500 kb	500–1000 kb	1000–1500 kb	>1500 kb	Total
AN	2714	295	41	10	3060
FJW	2304	436	89	28	2857
JW	3213	608	109	39	3968
JYS	1975	350	73	26	2424
KD	2489	378	74	23	2963
LWB	2173	356	62	17	2608
MXN	2964	270	23	3	3259
NZW	2610	365	61	13	3049
REX	2708	249	24	3	2984
SCW	2142	430	96	35	2703
WHHL	3122	662	134	40	3955

**Table 4:** Description of genes related to body size, growth, and development shared between rabbits and other animals.

Genes	Performance in Functional Assay
*INSIG2*	Body size [35]; body weight [36]; body mass index [37]
*GLI3*	Body weight [38]; body height [39]; skeletal development [40]
*ACOXL*	Back fat [41]
*PRKCQ*	Residual feed intake [42]; body weight [43]
*MAP3K13*	Muscle growth [44]
*BCL6*	Subcutaneous adipose tissue development [45]
*NRXN3*	Waist circumference [46]; body mass index [47]; obesity [48]
*LGR4*	Obesity [49]; body weight [50]

**Table 5:** Description of genes related to fertility shared between rabbits and other animals.

Genes	Performance in Functional Assay
*PLCB1*	Sexual maturity [51]; external genitalia development [52]
*GLI3*	Gonadal development [53,54]
*IL6R*	Prostaglandin E2 production [55]
*SST*	Fertility gene expression [56]
*EDNRA*	Fertility [57]
*TGFB2*	Gonadal development [58]
*ACOXL*	Reproductive traits [59]
*ITSN2*	Follicle development [60]
*PICALM*	Sperm release process [61]

**Table 6:** Description of genes related to resistance and immunity shared between rabbits and other animals.

Genes	Performance in Functional Assay
*PLCB1*	Heat stress adaptation [62]; thermo-tolerance [63]
*GSK3B*	Adaptation to hypoxic conditions [64]
*ISL1*	Plateau adaptation [65], myocardial development [66]
*PLD1*	Autophagy [67]; host cell invasion [68]
*LAP3*	Autophagy [69]
*CD86*	Immune regulation [70]
*SEC31A*	T cell immunity and immune tolerance [71]

## Data Availability

The sequencing data used for analysis are available at NCBI (PRJNA1011829), Detailed information regarding the data can be found in the corresponding dataset description at https://doi.org/10.1101/2024.03.26.586758.

## Supplementary Materials

The following supporting information can be downloaded at: https://www.mdpi.com/article/10.3390/genes15040433/s1. Table S1: Inbreeding Coefficients of Rabbits Based on Runs of Homozygosity (ROH); Table S2: Distribution of minor allele frequencies (MAF); Table S3: F_ST_ value between populations; Table S4: CV error for different K values; Table S5: GO classification and KEGG enrichment of the selected genes between white-coat and non-white-coat rabbits; Table S6: GO classification and KEGG enrichment of the selected genes between black rabbits and other rabbits rabbits; Table S7: GO classification and KEGG enrichment of the selected genes between angora and other rabbits; Table S8: GO classification and KEGG enrichment of the selected genes between small-body and large-body rabbits; Table S9: GO classification and KEGG enrichment of the selected genes between of sexual precocity rabbits; Table S10: GO classification and KEGG enrichment of the selected genes between Chinese indigenous rabbit and others.
